# Targeting the Human Cerebellum with Transcranial Direct Current Stimulation to Modulate Behavior: a Meta-Analysis

**DOI:** 10.1007/s12311-017-0877-2

**Published:** 2017-08-07

**Authors:** Viola Oldrati, Dennis J. L. G. Schutter

**Affiliations:** 1 Department of Brain and Behavioral Sciences, University of Pavia, Piazza A. Botta 6, 27100 Pavia, Italy; 2 Donders Institute for Brain, Cognition and Behaviour, Radboud University, Montessorilaan 3, 6525 HR Nijmegen, The Netherlands

**Keywords:** Cerebellum, Cognition, Meta-analysis, Motor, Performance, Transcranial direct current stimulation

## Abstract

Transcranial direct current stimulation (tDCS) is increasingly used to study motor- and non-motor-related functions of the cerebellum. The aim of the present study was to quantitatively review available studies to estimate the efficacy of cerebellar tDCS in altering motor- and cognitive-related behavioral performance in healthy volunteers. The present meta-analysis included 32 sham-controlled studies. Results from random effects modeling of the cumulative effect size demonstrated that anodal and cathodal tDCS to the cerebellum were effective in changing performance. No evidence for polarity-dependent effects of cerebellar tDCS was found. Current findings establish the feasibility to target motor and non-motor-related cerebellar functions with tDCS, but arguably due to anatomical differences between the cerebellum and cerebral cortex, the polarity of tDCS is not predictive of the direction of the behavioral changes in healthy volunteers.

## Introduction

The observation that exogenous weak electric direct currents (DC) applied to the primary motor cortex (M1) have polarity-dependent effects on corticospinal excitability introduced novel opportunities in the field of non-invasive brain stimulation to study brain-function relations [1].

Nowadays, transcranial DC stimulation (tDCS) is routinely used as a means to modulate and study functions of the cerebral cortex in the healthy and pathological brain [2]. The biophysical mechanism underlying the effects of tDCS is proposed to involve polarization of superficial nerve tissue that increases spontaneous neuronal firing rates during anodal stimulation and decrease spontaneous neural firing rates during cathodal stimulation [3–5].

Neurophysiological evidence for the ability to modulate cerebellar function using transcranial electric current comes from previous work that administered single high-voltage transcranial electric stimuli across the base of the skull and modulated activity in the dentate-thalamo-cortical pathway to M1 [6, 7]. In more recent studies, administering weak electric direct currents over the posterior fossa also showed to be effective in modulating cerebellar output [8, 9]. For example, cerebellar tDCS has shown to interfere with motor cortex synaptic plasticity during paired associative stimulation involving median nerve and motor cortex transcranial magnetic stimulation. This finding indicates that the cerebellum may be involved in the synchronization of sensory input and motor output [9]. In another study, Galea and colleagues [8] reported polarity-dependent effects of cerebellar DC stimulation (cDCS), similar to cerebral cortical DC stimulation. Paired-pulse cerebello-cortical transcranial magnetic stimulation was used to demonstrate an increase of M1 excitability following cathodal cDCS, whereas a reduction of M1 excitability was measured after anodal cDCS. The polarity-dependent effects concur with the idea of a respective decrease and increase of Purkinje cell-mediated inhibition of M1 [8]. In further support of the physiological data, computational modeling studies have confirmed that exogenous weak electric currents at an intensity of 2 mA can reach the outer layers of the cerebellar cortex [10]. The possibility to non-invasively target the human cerebellum with tDCS introduces new opportunities to study its role in motor and also non-motor functions [11].

In spite of the available evidence, several issues that include the scalp-to-cerebellum distance, limited spatial resolution of tDCS, and the unknowns associated with the effects of exogenous direct currents at the cellular level can cause considerable variance in the extent to which DC stimulation is consistent in effectively modulating the cerebellum. Furthermore, whether cDCS has similar anodal-cathodal polarity-dependent effects as observed for DC stimulation to M1 is still an open question [8]. Particularly, since the effects of tDCS likely depend on the nature of the cerebellar process contributing to the behavior under study [12], inferences about anodal cDCS causing functional enhancement and cathodal cDCS leading to functional disruptions remain speculative. Together with the existing skepticism on the reliability of tDCS effects, we performed a meta-analysis of sham-controlled studies to examine the effects of tDCS to the cerebellum on motor and non-motor functions in healthy volunteers. The aim of the present study was twofold: (1) Assess the efficacy and reliability of cDCS to induce behavioral effects; (2) test the hypothesis that anodal cDCS improves and cathodal cDCS impairs performance.

## Material and Methods

### Study Selection

**Fig. 1:**
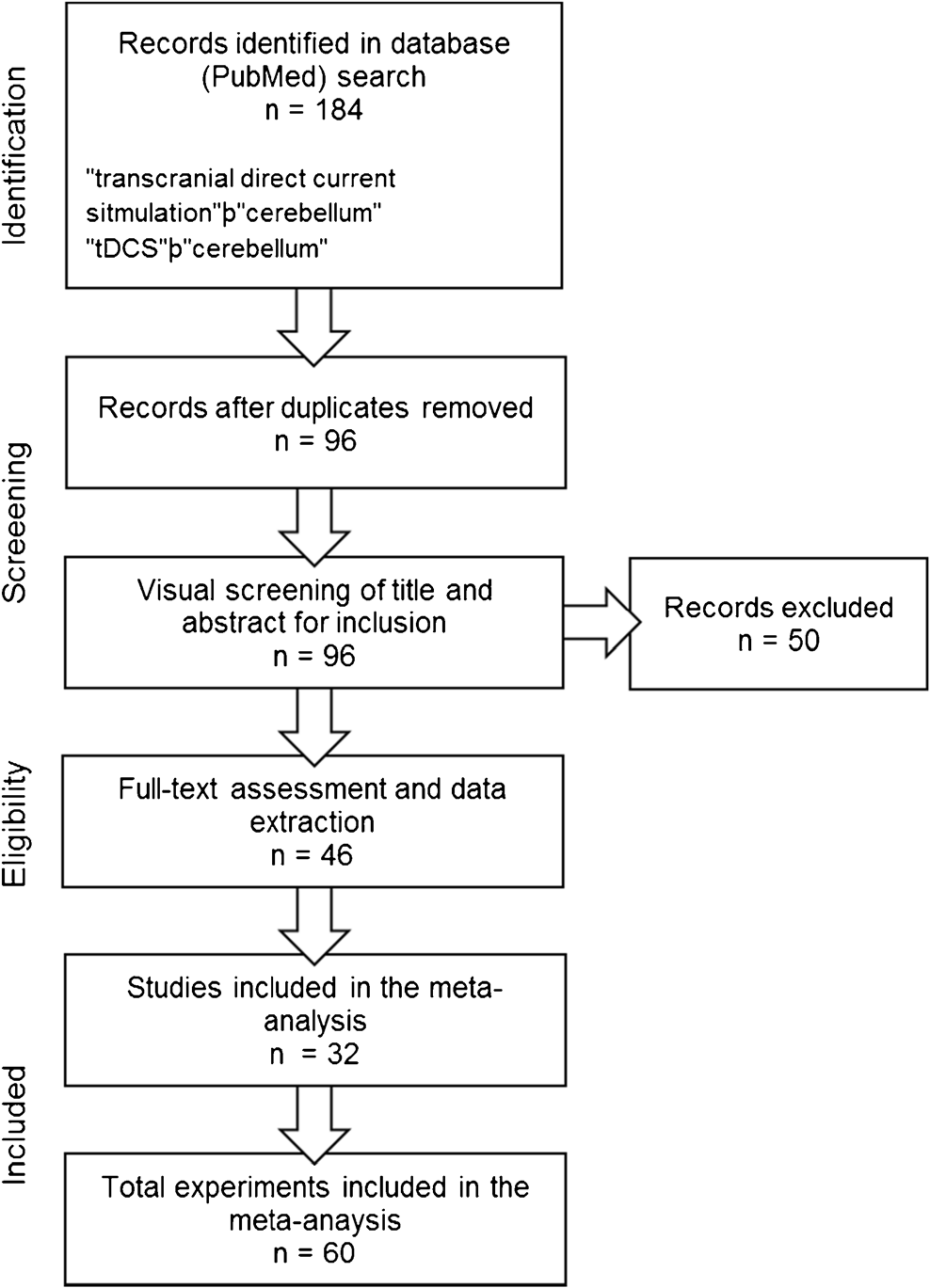
Flowchart study selection procedure

A literature search was conducted using the scientific online database PubMed to identify potential studies for inclusion in the meta-analysis in the period between January 2000 and March 2017. Search criteria were “transcranial direct current stimulation”þ“cerebellum” and “tDCS”þ“cerebellum.” In addition, the reference lists of previous reviews [12, 13] were screened to minimize the risk of overlooking potentially suitable studies for inclusion. Studies that met the following criteria were included: (i) adult healthy volunteers, (ii) sham-controlled randomized experimental design, (iii) administration of tDCS with at least one electrode placed over the cerebellum, (iv) cognitive or motor performance index (i.e. accuracy or reaction times) as primary endpoint, (v) article published in a peer-reviewed English-language journal, and (vi) study approved by a medical ethical committees or review board. In Fig. 1, the flowchart of the selection procedure is presented.

The literature search identified a total of 184 articles. After the completion of study retrieval and removal of duplicates, title and abstract of the 96 records remained were screened against inclusion/exclusion criteria. Sixty articles were selected to undergo full-text examination for eligibility, and an additional 18 studies were excluded. The remaining 32 studies were included in the present meta-analysis. Table 1 shows an overview of the main characteristics of the selected studies. Of these 32 studies, 15 studies applied both anodal and cathodal stimulation (ID: 4, 10, 14, 19, 21, 22, 24, 27, 28, 29, 31, and 32) [17, 23, 27, 30, 32, 34–36, 39–41, 43, 44], 14 anodal (ID 1, 5, 6, 7, 8, 11, 12, 13, 15, 16, 18, 20, 23, and 30) [14, 18–21, 24–26, 29, 31, 33, 42, 45], and three cathodal tDCS (ID: 3, 9, and 26) [16, 22, 38] (Table 1). In study 2 [15] were reported three experiments, each one comparing anodal and cathodal stimulation. Study 17 [28] includes seven experiments that applied only anodal stimulation. Study 22 [35] reports two experiments where both anodal and cathodal stimulations were used. In experiment 25, both anodal and cathodal stimulations were applied, whereas only anodal stimulation was applied in the second experiment (ID: 22) [37]. Except for study 1 [14] that consisted of two stimulation sessions per condition, and study 4 [17] in which participants underwent three stimulation sessions per condition, all studies administered one stimulation session per condition.

**Table 1:** Study characteristics

ID	Study	Exp.	Design	tDCS type	On/offline	*N* (real–sham)	Active electrode	Reference electrode	Electrodes size	Intensity	Duration	Sham control	Domain	Function	Task execution	Measure	Hedges’ *d*	
																	Anode	Cathode
1	Avila et al. [14]	1	Within	Anodal	Online	14–14	Right cerebellar hemisphere	Left BM	1.2 cm^2^–1.2 cm^2^	1.5 mA	15 min	30 s	Motor	Saccade adaptation	During cDCS	Accuracy	0.24	x
2	Beyer et al. [15]	2	Between	Anodal/cathodal	On/offline	10–10	Right cerebellar hemisphere	Right DM	35 cm^2^–35 cm^2^	2.0 mA	20 min	40 s	Motor	Conditioned eyeblink	cDCS off: trial 1–16, on: trial 17–50, off: trial 51–100	Accuracy	−1.28	−1.34
		3	Between	Anodal/cathodal	On/offline	10–10	Right cerebellar hemisphere	Right BM	36 cm^2^–35 cm^2^	2.0 mA	20 min	40 s			cDCS off: trial 1–16, on: trial 17–50, off: trial 51–100	Accuracy	−0.26	2.19
		4	Between	Anodal/cathodal	Online	10–10	Right cerebellar hemisphere	Right BM	37 cm^2^–35 cm^2^	2.0 mA	20 min	40 s			During cDCS	Accuracy	0.39	0.04
3	Boehringer et al. [16]	5	Within	Cathodal	Offline	40–40	Right cerebellar hemisphere	Right BM	25 cm^2^–25 cm^2^	2.0 mA	25 min	30 s	Cognition	Verbal working memory	Immediately after cDCS	Accuracy	x	−0.35
4	Cantarero et al. [17]	6	Between	Anodal/cathodal	Offline	11–11	Right cerebellar hemisphere	Right BM	25 cm^2^–25 cm^2^	2.0 mA	20 min	30 s	Motor	Motor learning	Prior, during, and immediately after cDCS	Accuracy	1.34	−0.1
5	Craig et al. [18]	7	Within	Anodal	On/offline	16–16	Vermis	Right BM	50 cm^2^–25 cm^2^	2.0 mA	20 min	30 s	Motor	Posture control	During, and 0 and 30 min after cDCS	Accuracy	0.29	x
6	D’Mello et al. [19]	8	Between	Anodal	Offline	18–14	Right cerebellar hemisphere	Right clavicle	35 cm^2^–35 cm^2^	1.0 mA	20 min	0 s	Cognition	Semantic prediction	7 min after cDCS	Accuracy	−0.37	x
7	Doppelmayr et al. [20]	9	Between	Anodal	On/offline	21–32	Vermis	Right posterior cerebral cortex^a^	3.14 cm^2^	1.0 mA	21 min	30 s	Motor	Motor adaptation	During, and 0 and 20 min after cDCS	Accuracy	0.06	x
8	Ehsani et al. [21]	10	Between	Anodal	On/offline	20–19	Right cerebellar hemisphere	Right DM	25 cm^2^–25 cm^2^	2.0 mA	20 min	60 s	Motor	Motor learning	During, and 35 min and 48 h after cDCS	Accuracy	−1.88	x
9	Fernandez et al. [22]	11	Between	Cathodal	Offline	14–14	Right cerebellar hemisphere	Right BM	35 cm^2^–35 cm^2^	2.0 mA	20 min	0 s	Motor	Motor adaptation	Immediately after cDCS	Accuracy	x	0.12
10	Ferrucci et al. [23]	12	Within	Anodal/cathodal	Offline	21–21	Vermis	Right DM	35 cm^2^–35 cm^2^	2.0 mA	20 min	10–15 s	Cognition	Emotion recognition	35 min after cDCS	Reaction times	0.14	−0.45
11	Ferrucci et al. [24]	13	Within	Anodal	Offline	21–21	Vermis	Right DM	35 cm^2^–35 cm^2^	2.0 mA	20 min	30 s	Cognition	Procedural learning	6 min after cDCS	Reaction times	−0.28	x
12	Galea et al. [25]	14	Between	Anodal	On/offline	30–30	Right cerebellar hemisphere	Right BM	25 cm^2^–25 cm^2^	2.0 mA	15 min	30 s	Motor	Motor learning	During and immediately after cDCS	Accuracy	−1.83	x
13	Hardwick et al. [26]	15	Between	Anodal	Online	11–11	Right cerebellar hemisphere	Right BM	25 cm^2^–25 cm^2^	2.0 mA	15 min	30 s	Motor	Motor learning	During cDCS	Accuracy	−2.69	x
14	Inukai et al. [27]	16	Within	Anodal/cathodal	Offline	16–16	Vermis	Forehead	35 cm^2^–35 cm^2^	2.0 mA	20 min	0 s	Motor	Posture control	Immediately after cDCS	Accuracy	0.15	0.05
15	Jalali et al. [28]	17	Between	Anodal	On/offline	14–14	Right cerebellar hemisphere	Right BM	25 cm^2^–25 cm^2^	2.0 mA	25 min	10 s	Motor	Visuomotor adaptation	During and immediately after cDCS	Accuracy	0.68	x
		18	Between	Anodal	On/offline	10–10	Right cerebellar hemisphere	Right BM	25 cm^2^–25 cm^2^	2.0 mA	25 min	10 s			During and immediately after cDCS	Accuracy	0.00	x
		19	Between	Anodal	On/offline	14–13	Right cerebellum	Right BM	25 cm^2^–25 cm^2^	2.0 mA	25 min	10 s			During and immediately after cDCS	Accuracy	0.00	x
		20	Between	Anodal	Online	12–12	Right cerebellum	Right BM	25 cm^2^–25 cm^2^	2.0 mA	25 min	10 s			Immediately after cDCS	Accuracy	0.47	x
		21	Between	Anodal	On/offline	18–18	Right cerebellum	Right BM	25 cm^2^–25 cm^2^	2.0 mA	25 min	10 s			During and immediately after cDCS	Accuracy	−0.05	x
		22	Between	Anodal	On/offline	16–16	Right cerebellum	Right BM	25 cm^2^–25 cm^2^	2.0 mA	25 min	10 s			During and immediately after cDCS	Accuracy	0.02	x
		23	Between	Anodal	On/offline	13–13	Right cerebellum	Right BM	25 cm^2^–25 cm^2^	2.0 mA	25 min	10 s			During and immediately after cDCS	Accuracy	0.02	x
16	Lametti et al. [29]	24	Between	Anodal	Online	17–18	Right cerebellum	Right BM	25 cm^2^–25 cm^2^	2.0 mA	15 min	30 s	Cognition	Speech perception	During and 7 min after cDC	Reaction times	−2.86	x
17	Macher et al. [30]	25	Within	Anodal/cathodal	Offline	16–16	Right cerebellum	Right BM	25 cm^2^–25 cm^2^	2.0 mA	25 min	30 s	Cognition	Verbal working memory	Immediately after cDCS	Accuracy	−0.62	0.02
18	Majidi et al. [31]	26	Between	Anodal	Online	10–10	Right cerebellum	Right BM	35 cm^2^–35 cm^2^	2.0 mA	20 min	30 s	Cognition	Probabilistic learning	during cDCS	Accuracy	−0.76	x
19	Miall et al. [32]	27	Between	Anodal/cathodal	Online	26–20	Right cerebellum	Right DM	25 cm^2^–25 cm^2^	2.0 mA	20 min	30 s	Cognition	Semantic processing	During cDCS	Accuracy	0.00	0
20	Panico et al. [33]	28	Between	Anodal	Offline	13–13	Right cerebellum	Right DM	25 cm^2^–25 cm^2^	2.0 mA	21 min	30 s	Motor	Motor adaptation	During cDCS	Accuracy	−0.20	x
21	Picazio et al. [34]	29	Within	Anodal/cathodal	Offline	13–13	Left cerebellum	Left DM	25 cm^2^–25 cm^2^	2.0 mA	20 min	45 s	Cognition	Visuospatial attention	11 min after cDCS	Accuracy	−0.48	−0.29
22	Pope and Miall [35]	30	Between	Anodal/cathodal	Offline	22–22	Right cerebellum	Right DM	25 cm^2^–25 cm^2^	2.0 mA	20 min	15 ms 110 μA every 550 ms	Cognition	Working memory	Immediately after cDCS	Accuracy	0.21	0.48
		31	Between	Anodal/cathodal	Offline	22–22	Right cerebellum	Right DM	26 cm^2^–25 cm^2^	2.0 mA	21 min	15 ms 110 μA every 550 ms	Cognition	Language	Immediately after cDCS	Reaction times	0.00	0.36
23	Samaei et al., [45]	32	Between	Anodal	On/offline	15–15	Right cerebellum	Right DM	25 cm^2^–25 cm^2^	2.0 mA	20 min	30 s	Motor	Motor learning	During, and 30 min and 48 h after cDCS	Accuracy	0.24	
24	Shah et al. [36]	33	Within	Anodal/cathodal	Offline	8–8	Left cerebellum	Left BM	8 cm^2^–35 cm^2^	1.0 mA	15 min	0 s	Motor	Visuomotor adaptation	10, 30, and 60 min after cDCS	Accuracy	1.12	1.53
25	Shimizu et al. [37]	34	Between	Anodal/cathodal	On/offline	23–21	Vermis	Left/right BM	35 cm^2^–35 cm^2^	2.0 mA	20 min	30 s 2.0 mA,20 min 1.0 mA	Motor	Motor learning	Immediately after cDCS	Accuracy	0.1	0.33
		35	Between	Anodal	On/offline	20–14	Vermis	Left/right BM	35 cm^2^–35 cm^2^	2.0 mA	20 min	30 s 2.0 mA,20 min 1.0 mA			During and immediately after cDCS	Reaction times	0.13	x
26	Spielmann et al. [38]	36	Within	Cathodal	Offline	24–24	Right cerebellum	Right DM	25 cm^2^–25 cm^2^	2.0 mA	20 min	30 s	Cognition	Language	Immediately after cDCS	Reaction times	x	0.39
27	Steiner et al. [39]	37	Between	Anodal/cathodal	On/offline	10–10	Vermis	Right-left BM	35 cm^2^–25 cm^2^	2.0 mA	10 min	20 s	Motor	Motor learning	Immediately after cDCS	Accuracy	0.44	0.27
28	Taubert et al. [40]	38	Between	Anodal/cathodal	On/offline	14–15	Right cerebellum	Right BM	25 cm^2^–25 cm^2^	2.0 mA	20 min	30 s	Motor	Motor learning	During and 24 h after cDCS	Accuracy	−0.80	−0.22
29	van Wessel et al. [41]	39	Within	Anodal/cathodal	Online	12–12	Right cerebellum	Left BM	1.2 cm^2^–1.2 cm^2^	2.0 mA	20 min	30 s	Cognition	Working memory	During cDCS	Accuracy	0.02	−0.11
30	Verhage et al. [42]	40	Between	Anodal	Online	20–19	Right cerebellum	Right BM	1.2 cm^2^–1.2 cm^2^	1.5 mA	20 min	60 s	Cognition	Implicit categorization	During cDCS	Accuracy	−0.07	x
31	Yavari et al. [43]	41	Between	Anodal/cathodal	Offline	13–13	Right cerebellum	Right BM	25 cm^2^–25 cm^2^	2.0 mA	15 min	30 s	Motor	Visuomotor adaptation	Immediately after cDCS	Accuracy	1.25	−0.67
32	Zuchowski et al. [44]	42	Between	Anodal/cathodal	Online	10–10	Right cerebellum	Right BM	35 cm^2^–35 cm^2^	2.0 mA	42.9 min	30 s	Motor	Motor adaptation	During cDCS	Accuracy	−1.52	1.50

*BM* right buccinator muscle, *cDCS* cerebellar direct current stimulation, *DM* deltoid muscle

^a^This study used high-density tDCS in which the Oz, O2, P8, and PO8 locations served as the return sites

### Data Synthesis and Analysis

Performance accuracy was our primary dependent variable of interest. Reaction times were used in case no data on accuracy were available. Experiments (ID: 1, 2, 4, 5, 7–12, 15–17, 19–28, and 30–32) that evaluated performance across multiple time points, the aggregate mean [(*μ*_1_ + *μ*_2_ + … + *μ*_*n*_^2^) / *k*] and pooled SD [√((SD_1_^2^ + SD_2_^2^ + … + SD_n_^2^) / *k*), where *k* = total number of data points, were calculated. The following descriptive data were taken from each study: sample size, mean, and standard deviation (SD) of the outcome measure for the stimulation and the sham condition. In case standard errors of the mean (SE) were provided, SD were calculated by applying the following formula SD = SE × √*n*. When data were presented graphically, mean and SD were estimated from the figures using free WebPlotDigitizer gms3.10 software (http://arohatgi.info/WebPlotDigitize). Corresponding authors were contacted in case the relevant numbers for the analysis could not be extracted from the paper.

The effect size metric Hedges’ *d* was used which is a standardized mean difference that accounts for the fact that the sampling variance for “active” and “sham” conditions may not always be equal [36]. From these effect sizes, the Hedges’ *d* values were calculated to correct for a bias in effect size due to small group samples [46]. For the meta-analysis, non-parametric variances were chosen to control for small sample sizes. Next, a weighted average was used to compute the cumulative effect size (*Ē*) and the 95% confidence intervals (CI). *Ē* represents the aggregated magnitude of the effect size of the included studies [46].

To address our first research question related to the efficacy and reliability of cDCS to induce behavioral effects, the unsigned cumulative effect sizes (|*Ē*|) were tested in a random effects model. Total heterogeneity of the effect sizes (*Q*_*T*_) was calculated and tested against the *χ* ^2^ distribution with (*n* - 1) degrees of freedom [47]. In addition, the *I* ^2^ [(*Q*_*T*_ − *df* / *Q*_*T*_) × 100] of heterogeneity was reported which is an index for the residual proportion of the observed variance if sampling error is zero [48].

A significant *Q*_*T*_ means that the variance of the effect sizes is greater than to be expected from sampling errors and suggests that the observed variance can be explained by other variables besides cDCS. To explore the robustness of the results to the possibility of publication bias, fail-safe number of studies was computed to obtain an estimate of how many non-significant or missing studies would render the observed meta-analytical results non-significant (Rosenthal’s method: *α* < 0.05). To address our second research question concerning the assumed polarity-dependent effects of cDCS, data were analyzed in a similar way as was done in the first series of analyses, except that the signed effect sizes (*Ē*) were used. All analyses were performed with MetaWin version 2 [49] and Wilson’s macros for meta-analyses in SPSS [50].

## Results

Unsigned cumulative effect size: The random effects model (*n* = 60) between real and sham cDCS shows a significant |*Ē*| of 0.55, 95%CI = 0.38–0.73, *Z* = 6.27, *p* < 0.001. Total heterogeneity was not significant, *Q*_*T*_ = 59.74, *p* = 0.45, *I* ^2^ = 1.23.

**Fig. 2:**
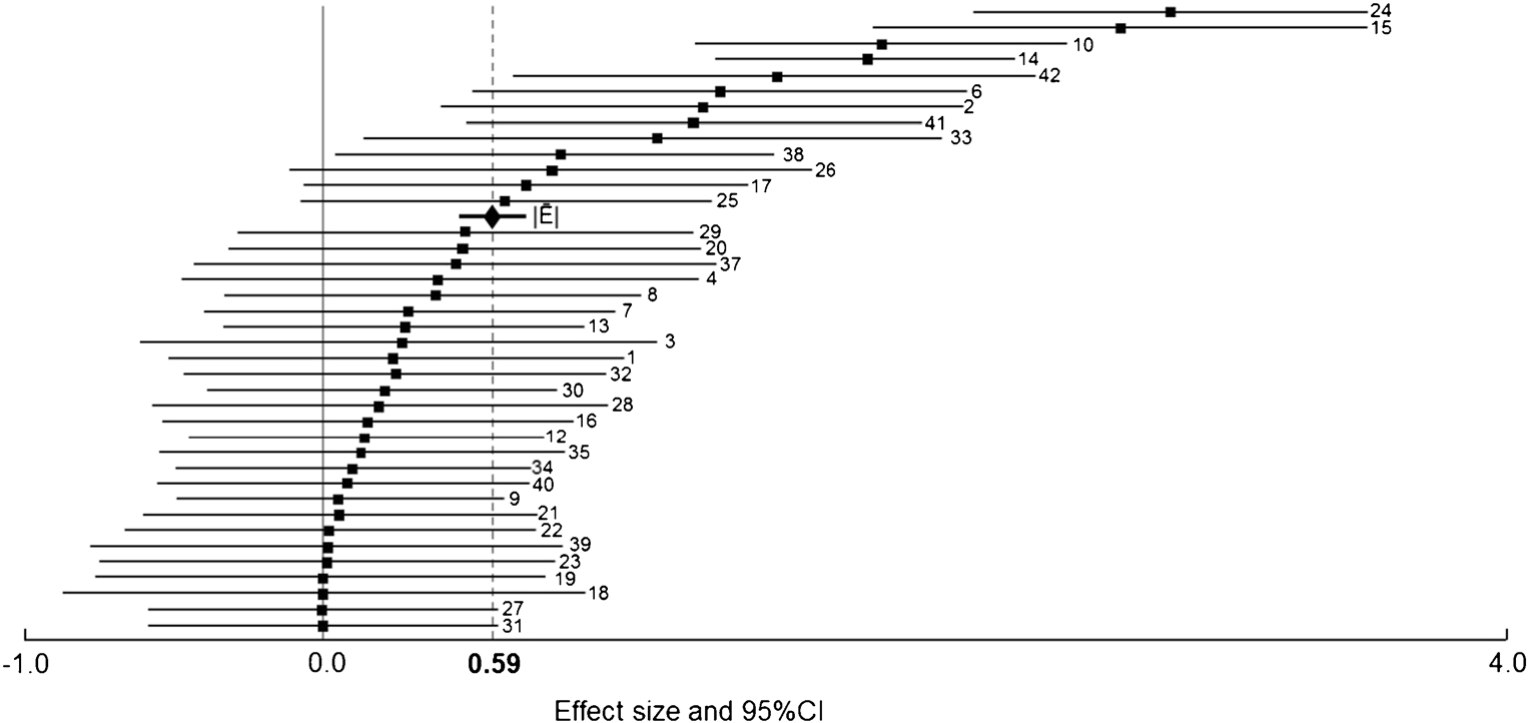
Forest plot showing effect size estimates (Hedges’ *d*) and 95% confidence interval of the experiments comparing anodal with sham cDCS in healthy volunteers

Anodal cDCS showed a significant |*Ē*| of 0.59, 95%CI = 0.34–0.83, *Z* = 4.84, *p* < 0.001 (Fig. 2). Total heterogeneity was not significant, *Q*_*T*_ = 35.94, *p* = 0.57, *I* ^2^ = 0, and the Rosenthal’s fail-safe number was 303.

**Fig. 3:**
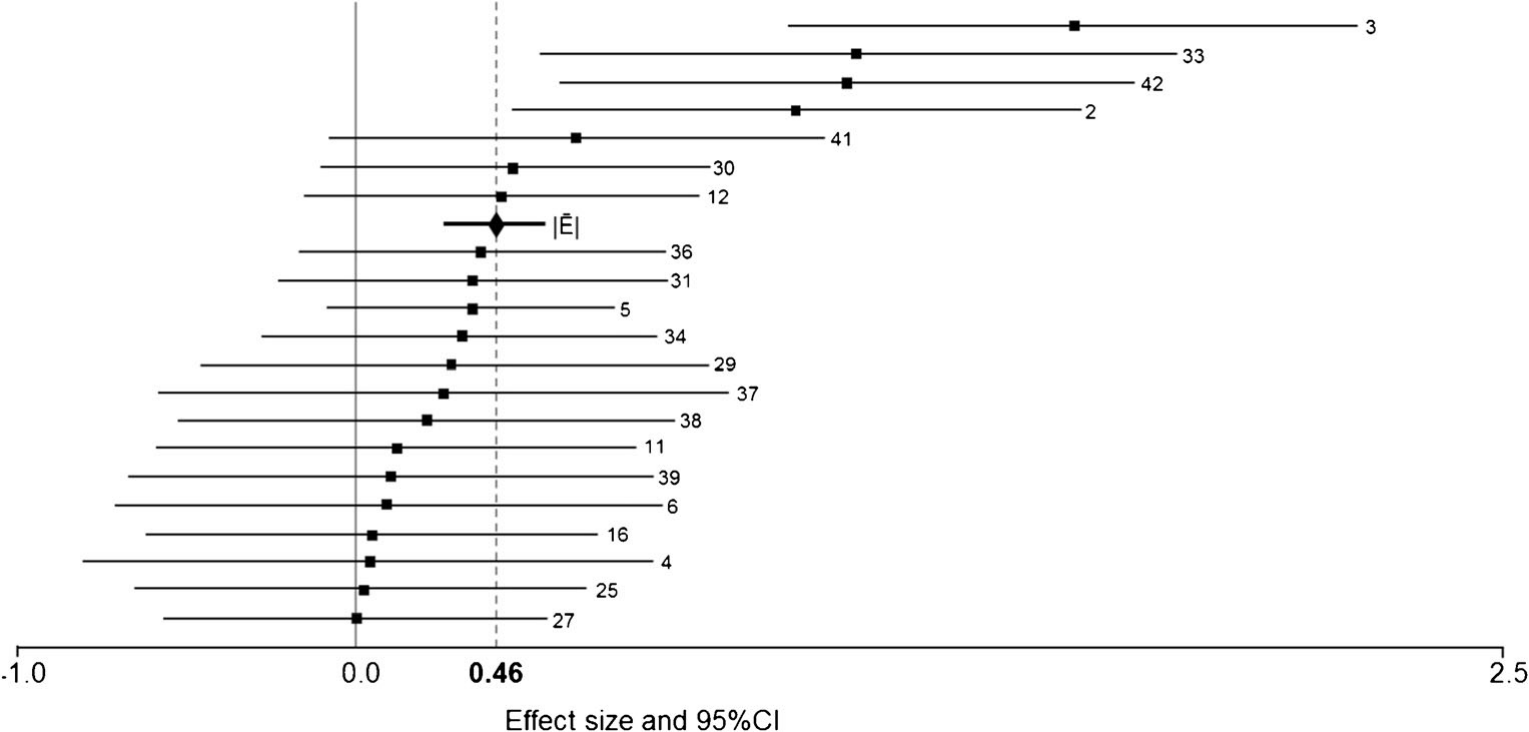
Forest plot showing absolute cumulative effect size estimates (Hedges’ *d*) and 95% confidence interval of the experiments comparing cathodal with sham cDCS in healthy volunteers

Cathodal cDCS showed a significant |*Ē*| of 0.46, 95%CI = 0.23–0.69, *Z* = 4.23, *p* < 0.001 (Fig. 3). Total heterogeneity was not significant, *Q*_*T*_ = 23.65, *p* = 0.26, *I* ^2^ = 15.43, and the Rosenthal’s fail-safe number was 131.

Even though effect sizes of anodal and cathodal cDCS were similar, *Q* = 0.31, *p* = 0.58, cDCS was more efficacious in modulating motor, |*Ē*| = 0.71, 95%CI = 0.49–0.92, than cognitive-related tasks, |*Ē*| = 0.32, 95%CI = 0.06–0.58, *Q* = 5.24, *p* = 0.02. Whether performances were measured during cDCS (online), |*Ē*| = 0.70, 95%CI = 0.32–1.07, after cDCS (offline), |*Ē*| = 0.61, 95%CI = 0.33–0.88, or both during and after cDCS (on-offline), |*Ē*| = 0.42, 95%CI = 0.14–0.69, did not influence the magnitude of the effect size, *Q* = 1.67, *p* = 0.43.

Signed cumulative effect size: The random effects model (*n* = 60) between real and sham cDCS showed a non-significant |*Ē*| of 0.55, 95%CI = 0.38–0.73, *Z* = 6.27, *p* < 0.001. Total heterogeneity was not significant, *Q*_*T*_ = 59.74, *p* = 0.45, *I* ^2^ = 1.23.

Anodal cDCS showed a non-significant *Ē* of 0.05, 95%CI = −0.22–0.31, *Z* = 0.30, *p* = 0.77. Total heterogeneity was not significant, *Q*_*T*_ = 47.40, *p* = 0.14, *I* ^2^ = 19.83.

Finally, the random effects model for cathodal versus sham cDCS was not significant, *Ē* = 0.15, 95%CI = −0.15–0.46, *Z* = 1.07, *p* = 0.29. Total heterogeneity was also not significant, *Q*_*T*_ = 24.86, *p* = 0.21, *I* ^2^ = 19.87.

In sum, 1–2 mA cDCS is effective in modulating motor- and non-motor-related performance, but there is no evidence for polarity-dependent effects of anodal and cathodal cDCS on behavioral indices of cerebellar functioning in healthy volunteers. Table 2 shows the main statistical outcomes of the meta-analysis.

**Table 2:** Main results

Random effects model		Functional domain	n	Effect size	95%CI		*Z*	*p* value	*Q*_*t*_	*p* value	*I* ^2^	Fail-safe number
					*Lower upper*							
|*Ē*|	Total		60	0.55	0.38	0.73	6.27	<0.001	59.74	0.45	1.23	844
		Motor	37	0.71	0.49	0.92	6.49	<0.001	33.85	0.57	0	330
		Cognition	23	0.32	0.06	0.58	2.39	0.02	15.13	0.85	0	148
	Anodal		39	0.59	0.34	0.83	4.84	<0.001	35.94	0.57	0	303
	Cathodal		21	0.46	0.23	0.69	4.23	<0.001	23.65	0.26	15.42	131
*Ē*	Total		60	−0.09	−0.31	0.13	0.78	0.44	63.61	0.32	7.81	-
	Anodal		39	0.05	−0.22	0.31	0.30	0.77	47.40	0.14	19.83	-
	Cathodal		21	0.15	−0.15	0.46	1.07	0.29	24.86	0.21	19.87	-

*CI* confidence interval, |*Ē*| unsigned cumulative effect size, *Ē* signed cumulative effect size

## Discussion

The feasibility of tDCS to modulate cerebellar functions has provided new opportunities to in vivo study cerebellar contributions in motor and non-motor-related function in healthy volunteers. In this study, we found that 1–2 mA of tDCS targeting the cerebellum is able to modulate cognitive and motor performance in healthy volunteers. Evidence for polarity-dependent effects of anodal and cathodal cDCS on improving and disrupting behavior respectively was not found.

Modeling studies suggest that the observed effects can be attributed to changes in cerebellar function. Estimations of electric field properties of bipolar DC stimulation using an inion-based cerebellum-buccinator muscle montage show that, due to the volume and homogenous structure of the cerebellum, the electric field distributions are more focused as compared to other montages targeting the cerebral cortex [43, 51]. Furthermore, lower input impedance of Purkinje cells that results in larger current flows through the cell membrane may sufficiently compensate for the low maximum electric field strength at the superficial part of the cerebellum in comparison to neurons in the cerebral cortex [10]. In line with the available neurophysiological evidence, our meta-analysis provides behavioral support for the view that weak DC can alter cerebellar functioning.

Analyses showed that motor performance was significantly more affected than cognitive performance. There may be several explanations for this finding. On one hand, this result may suggest that the cerebellum plays a more central role in motor- as compared to non-motor-related functions. This is in line with the classical view of the cerebellar functions. However, our analyses do provide reliable evidence that cognitive performance is also significantly affected, indicating that the cerebellum is not exclusively related to the motor function [52–54]. A more methodologically oriented explanation for the discrepancy in effects between motor and cognitive tasks may be due to differences in the sensitivity of the task and the dependent variable of interest to detect reliable changes in performance.

Despite these converging lines of evidence, biophysical mechanisms that can account for the effects of weak static electric fields on cerebellar physiology and behavior remain elusive [5, 12]. Moreover, substantial individual variability in anatomy as well as neurophysiological constitution plays a critical role in the efficacy of cDCS. This may also in part explain why, in contrast to the popular view of polarity-dependent effects of tDCS, neither anodal nor cathodal cDCS predicted the respective enhancement or impairment in behavior. A factor presumed to be involved may concern the unknowns regarding the degree to which the cerebellum is actively engaged in a particular motor task or cognitive function. For example, if the cerebellum does not play a role in a given neural processing stream, then anodal cDCS may increase spontaneous firing rates in the cerebellum. As a result, unrelated signals are introduced in the brain that may actually interfere with neural processing, causing functional impairments rather than improvements. In addition, the inhibitory-excitatory nature and timing of its contributions may be important for understanding the effects of a uniform electric field on cerebellar tissue. In fact, this issue may even be more critical when cDCS is delivered simultaneously during performance. At this point, one can only speculate about the functional role of the cerebellum in non-motor-related behavior. According to the universal cerebellar transform hypothesis, the cerebellum integrates internal and external information to optimize performance according to context [55]. The integration is suggested to reflect a coordinated sequence of processes associated with thought and action that occurs in the cortico-pontine-cerebellar-thalamo-cortical circuit [55]. Electric stimulation of the cerebellum appears a feasible technique to further examine the universal cerebellar transform hypothesis. However, in order to be able to better understand and predict the behavioral effects of cDCS, unraveling the working mechanisms of the cerebellum on the molecular, structural, and system’s level is required.

Even though the main outcomes of the meta-analyses support the view that DC stimulation is a viable approach to investigate cerebellar functions, the variability in outcome measures as well as the applied stimulation parameters across studies should be taken into account. In particular, our results do not unequivocally establish that cDCS is effective in all circumstances for reasons we discussed earlier. However, our data do show that low electric currents applied to the surface of the scalp can produce effects that may shed light on the contributions of the cerebellum in motor- and non-motor-related performance, but that is difficult to predict the direction of the effects relying on current polarity. Further research on how weak direct currents establish effects on cerebellar tissue and influence functional processes may help to improve cDCS designs.

Finally, several limitations of the present study should be mentioned. The limited number of studies available for our analysis prevented us from performing sub-analyses to explore questions such as under which circumstances polarity-dependent effects may still be applicable to cDCS and which types of function are particularly sensitive to cDCS. Also, our findings do not provide mechanistic insights into the cerebellar workings, and adding neuroimaging techniques to future studies seems a logical next step.

In conclusion, DC stimulation is effective in modulating cerebellar function, but no support is found for polarity-dependent effects of anodal and cathodal cDCS on enhancing and disrupting behavior, respectively.
